# Immunostimulation of Asian elephant (*Elephas maximus*) blood cells by parapoxvirus ovis and CpG motif-containing bacterial plasmid DNA upregulates innate immune gene expression

**DOI:** 10.3389/fimmu.2024.1329820

**Published:** 2024-03-25

**Authors:** Jonathan Haycock, Tanja Maehr, Akbar Dastjerdi, Falko Steinbach

**Affiliations:** ^1^ Faculty of Health and Medical Sciences, University of Surrey, Guildford, United Kingdom; ^2^ Department of Virology, Animal and Plant Health Agency, Addlestone, United Kingdom

**Keywords:** EEHV, elephant, innate immunity, zelnate, zylexis

## Abstract

The immune system of Asian elephants (Elephas maximus) is poorly studied, compared to that of livestock, rodents or humans. The innate immune response has become a focus of interest in relation to Elephant endotheliotropic herpesviruses (EEHVs). EEHVs cause a fatal hemorrhagic disease (EEHV-HD) and are a significant threat to captive Asian elephant populations worldwide. Similar to other herpesvirus infections, nearly all animals become infected, but only some develop disease. As progression to EEHV-HD is often acute, a robust innate immune response is crucial to control EEHV infections. This is invariably true of the host in the first instance, but it can also potentially be modulated by intervention strategies. Here, two immunostimulant veterinary medicinal products, authorized for use in domestic species, were tested for their ability to induce innate anti-viral immune responses in Asian elephant blood cells. Sequence data were obtained for a range of previously unidentified Asian elephant immune genes, including C-X-C motif chemokine ligand 10 (CXCL10), interferon stimulated gene 15 (ISG15) and myxovirus GTPase 1 (Mx1), and were employed in the design of species-specific qPCR assays. These assays were subsequently used in analyses to determine fold changes in gene expression over a period of 24 hours. This study demonstrates that both immunostimulant medications are capable of inducing significant innate anti-viral immune responses which suggests that both could be beneficial in controlling EEHV infections in Asian elephants.

## 1 Introduction

The activation of the vertebrate innate immune system centers on the detection of pathogen-associated molecular patterns (PAMPs) by pattern recognition receptors (PRRs) (1). Nucleic acid-sensing PRRs have been shown to confer resistance to infections at cellular level through the initiation of innate immune responses and particularly by stimulating the interferon (IFN) system (2, 3). Toll-like receptors (TLRs) 3, 7/8 and 9 recognize double-stranded ribonucleic acid (dsRNA), single-stranded ribonucleic acid (ssRNA) and DNA containing unmethylated CpG motifs respectively (4). Further, the stimulator of interferon genes (STING) cyclic GMP-AMP (cGAMP) synthase (cGAS) pathway is known to play a key role in the response to intracellular DNA (5–7), e.g. initiating the transcription of IFNs and proinflammatory cytokines in response to herpes simplex virus (HSV)-1 infections both *in-vivo* and *in-vitro* (8, 9).

Previously, the concept of paramunity, i.e. the non-specific but regulated stimulation of the innate immune system, had been proposed in relation to the use of inactivated parapoxvirus ovis (iPPVO) in veterinary models of disease (10–12). Multiple studies have suggested benefits of the veterinary application of iPPVO (currently marketed as Zylexis^®^; Zoetis, Leatherhead, UK), particularly in relation to viral diseases (13, 14). For example, reducing the severity of clinical signs caused by equine herpesviruses 1 and 4 (EHV1 and EHV4) in young horses (15). Further studies in human immune cells (16) and rodent models of HSV-1, HSV-2 and Aujeszky’s disease (17, 18), led to the conclusion that iPPVO stimulates not only the interferon system but also the early release of T-helper type 1 (Th1) cytokines such as IFNγ to limit infections *in vivo* (14, 19).

Unmethylated CpG motifs are nucleotide sequences serving as distinct PAMPs (20) that are also highly capable of stimulating the innate immune system in an array of species (21, 22). The protective role of CpG motifs has been previously reported in mice, where the intravaginal treatment of HSV2-infected mice provided significant protection against normally fatal disease, even if administered after infection (23). More generally, the use of CpG motifs as immunostimulants in infectious disease has been suggested in situations of predictable and imminent exposure to infections (24).

Recently, Zelnate^®^ (Bayer, Leverkusen, Germany) has been authorized in cattle for prophylactic and interventional use, consisting of CpG motif-containing bacterial plasmid DNA within a cationic liposome capsule (25, 26). It was originally assumed to stimulate bovine TLR-9, as described for CpG motifs in humans and mice (27–29), but more recent work concluded the downstream effects were mediated by the STING/cGAS pathway (30). Ultimately, both paramunity (to aid vaccination against an unrelated pathogen) and the use of CpG motifs directly relate to the relatively recently defined concept of trained immunity (31, 32).

Elephant endotheliotropic herpesviruses (EEHV) (33, 34), are the cause of a hemorrhagic disease (EEHV-HD) in juvenile Asian elephants between the ages of 1 and 8 years old (35). Whilst considerable advances have been made in the detection of EEHV-HD, mortality remains high due to the lack of efficacious antiviral therapies or vaccines (36, 37). This has driven recent research into alternative areas, including the role of the Asian elephant immune system, to identify means by which it might be manipulated to treat and potentially prevent EEHV-HD (38, 39). In this study, the effects of Zylexis and Zelnate on Asian elephant white blood cells were analyzed using a set of novel qPCR assays to determine immunological mechanisms underlying their potential clinical applications, specifically in the context of early EEHV infections.

## 2 Materials and methods

### 2.1 Animals and sample collection

The ten Asian elephants (eight adults and two juveniles) included in this study were housed at zoological collections within the United Kingdom. The eight adult elephants ranged from 16 to 35 years of age. The two juvenile elephants were 3 and 8 years old, respectively. Blood samples were collected into tubes containing ethylenediaminetetraacetic acid (EDTA) for the purpose of routine EEHV monitoring and/or diagnostic investigations according to routine veterinary practices. Veterinary interventions such as blood sample collection for the purpose of clinical health screening fall under the Veterinary Surgeons Act 1966 and do not require further ethical approval, as they are intended solely to maintain animal welfare. All animals were considered clinically healthy at the time of sample collection. Samples were sent at ambient temperature overnight to the laboratory and refrigerated at 4-8°C until being processed within 2 hours.

### 2.2 Asian elephant mRNA sequences and qPCR assay development

#### 2.2.1 Extraction of genomic DNA

Extraction of genomic DNA (gDNA) from 140 µL aliquots of whole EDTA blood, collected from a 16-year-old captive-born male elephant, was performed using the QIAamp Viral RNA Mini Kit (Qiagen, Manchester, UK) according to the manufacturer’s instructions and samples were eluted into a final volume of 60 µL of buffer AVE.

#### 2.2.2 Sample lysis for RNA extraction

Aliquots of 250 µL of whole EDTA blood were homogenized by repeated vigorous pipetting in 750 µL of TRIzol™ LS Reagent (Fisher Scientific, Loughborough, UK) and incubated at room temperature for five minutes. Each sample mixture was transferred to a Phasemaker™ Tube (Fisher Scientific) and 0.2 mL of chloroform (Sigma Aldrich, Gillingham, UK) was added. Tubes were shaken vigorously by hand for at least two minutes and then incubated for a further 15 minutes at room temperature. Following centrifugation at 4°C for five minutes at 15,000 x *g*, 300 µL of the colorless upper aqueous phase was transferred to a new tube.

#### 2.2.3 Silica membrane-based RNA extraction

RNA was extracted from the aqueous phase of lysis using the RNeasy^®^ MinElute^®^ Cleanup Kit (Qiagen, Manchester, UK) according to the manufacturer’s instructions and eluted into 12 µL of RNase-free water. The eluate was subject to in-solution DNase digestion using the RNase-free DNase Set (Qiagen) according to the manufacturer’s instructions. The removal of DNase enzyme was achieved with a final round of the RNeasy MinElute Cleanup kit as described above but with an additional 80% ethanol wash step immediately prior to sample drying and elution, based on the presence of contaminants in preliminary studies. Eluted RNA originating from the same blood sample was pooled, and the concentration and quality of eluted RNA was measured using a Nanodrop 2000 spectrophotometer (Agilent Technologies, Stockport, UK). Extracted RNA samples were stored at -80°C until further processing.

#### 2.2.4 First strand complimentary DNA synthesis

For each reverse transcription reaction, 250 ng of RNA was mixed in a nuclease-free microcentrifuge tube with 500 ng of random hexamers, 1 µL of 10 mM deoxyribonucleotide triphosphate (dNTP) mix (both Promega UK Ltd., Southampton, UK) and sterile, nuclease-free water to a final volume of 13 µL. The mixture was heated to 65°C for five minutes, incubated on ice for at least one minute and briefly centrifuged. A mixture of the following reagents was then added: 200 units of Invitrogen™ Superscript™ III Reverse Transcriptase (Fisher Scientific), 40 units of recombinant RNasin^®^ ribonuclease inhibitor (Promega UK Ltd.), 1 µL of kit-supplied 0.1 M dithiothreitol (DTT) and 4 µL of kit-supplied 5x First-Strand Buffer. The contents of the tube were mixed by gentle pipetting and the reaction was incubated at 25°C for five minutes. The incubation temperature was increased to 50°C for 60 minutes before the reaction was inactivated by heating at 70°C for 15 minutes. The resulting complimentary DNA (cDNA) was pooled from technical replicates, diluted 1:3 in Endotoxin-free Buffer TE (10 mM Tris-HCl; 1 mM EDTA; pH 8.0; Qiagen) based on preliminary experiments demonstrating the low-level presence of PCR inhibitors, and stored at -20°C.

#### 2.2.5 Identification of Asian elephant mRNA sequences

The DNA sequence reads from six Asian elephant genomes available at the time via the NCBI SRA database (https://www.ncbi.nlm.nih.gov/sra; accession numbers SRS927124, SRS927123, SRS927126, SRP065915, SRS1158889, ERS365881 and ERS365882 (40, 41); were used in combination with the online NCBI Basic Local Alignment Search Tool (BLAST; https:blast.ncbi.nlm.nik.gov/Blast.cgi) for the prediction of mRNA sequences for the following Asian elephant genes: elongation factor 1 alpha (*EF1α*), *IFNω*, C-X-C motif chemokine ligand 10 (*CXCL10*), granulocyte-macrophage colony-stimulating factor (*GMCSF*), *IL1β*, *IL6*, *IL8*, IFN regulatory factor (*IRF*)3, *IRF7*, *IRF9*, IFN stimulated gene 15 (*ISG15*), myxovirus GTPase 1 (*Mx1*), NFκB subunit 2 (*NFκB2*), 2’-5’-oligoadenylate synthetase 1 (*OAS1*), double-stranded RNA-dependent protein kinase (*PKR*), signal transducer and activator of transcription 1 (*STAT1*), *STAT2*, *TLR3* and *TLR9*. For each gene, the corresponding published mRNA sequences of human (*Homo sapiens*) and the published predicted mRNA sequences of African elephant (*Loxodonta africana*) were used as respective templates in predicted sequence assemblies. Where possible, the 5’- and 3’- untranslated regions (UTRs) immediately upstream of the initial start codon and downstream of the final stop codon, respectively, were included in sequence predictions.

Amplification and sequencing primers were initially designed against the 5’ and 3’ ends of available Asian elephant predicted sequence data for each gene. This included the UTRs outside of the 5’ start codon and 3’ stop codon to facilitate amplification of entire gene sequences. Subsequent design of additional primers was required for several genes to complete the initial sequence data. All amplification and sequencing primers used in this study were synthesized by Eurofins Genomics (Ebersburg, Germany; shown in **Table 1**).

**Table 1:** cPCR and sequencing primers used to amplify Asian elephant innate immune genes.

Target Gene	F primer (5’-3’)	R primer (5’-3’)
*CXCL10*	CTCAGCTGTGTGCCCACATT	ATAGGGAAGTGATGGCAGAG
*EF1α*	GGTGTTGGTGAAAACTATCGC	GACCGTTCTTCCACCACTG
*GMCSF*	GAAAGGCTAAAGTCCTCAGGAG	CAGTCAAAGGGAATGATGTGCAG
*IFNω*	AGCCGTGATAGAAAGAATGTCTTC	CCAAAGATATGCGTGCAAGTGC
*IL1β*	CTCTTCTCTTTACATAGGTTTCTG	TCTATTCCCTTTCTGCCAGCC
*IL6*	TTCAGCCCACCAGGAACAAA	TTGACCAGCTGAAAGAATGCC
*IL8*	CACACAAGTGTCTAGGACAAG	CATTGGCATCTTCACTGAGGTA
*IRF3*	GTCTTGGCCTCAGTCCCAG	TCCTGCAGATAGGCCTTGTAC
*IRF7*	GGCTGGAAAACTAACTTCCGCT	TGTAGCGAAGCTGCTTCTGGT
*IRF9*	GCATGCAAGCAAGCAAGACTTC	TTTGAAGAGCTCCACGCACTC
*ISG15*	CTGCTTCCTGAGCCACTTGATG	CAGAAAACTCAGCGTCCCCTG
*Mx1*	GGCTTCATTGACAAAGGAGGAA	ACGGGGCTGAGCAGAGA
	GGGCGTGGAACAGGACCT	CCATTTGAGGAACTCACTTCGG
	TGCCCGCCATCGCTGTCAT	CTGGCAGTAGACAATCTGCTC
	GAAGGTGGTGGATGTGGTGC	
*NFκB2*	TGGACCTGGTAACACACAGTGA	GCCAAGGAGGAAGGGAAAAAG
*OAS1*	AGACTAACAGGTCCCAGGC	GATGACTCTGGAGCCCAGT
*PKR*	GCAGGTTTCTTCATGGAGGAAC	TGTGTTTTGCTTTGAAAACGTCGC
	TCAGGTGGATATGGCGACGTTT	ATGTATCGCCATGTTCCCTTGC
*STAT1*	GAACTTACCCAGAATGCCCTC	AACTGCCAACTCAGCACCTCT
*STAT2*	GCAGTTCTCGTCGTACATTGG	TGGTGGATGATTTCAGTCAGCG
*TLR3*	CTCTATAGAACAAGCAATATGACTTT	TTAAACTTGGAAAGTTACTCCTTTGT
	CAGCTCCTTTACGAGTTTGCCAA	GCCGCATATTCAAACTGCTCTG
	ATCAGCCTGCAAAGACAGTGC	GGTATCTCACATTGAGCAGCC
	CCCTGATGAAAGCCTTTGGATTG	
	CTGCGTGAATTTGACAGAACTCC	
*TLR9*	CAACTGCATCACCAAAACCGTG	GTGGGAATAGAGGTCTCCTTCA
	CATAGGCCACAACCTCAGCTTT	CTAGCACAAACAGCGTCTTGC

For amplification of these Asian elephant mRNA sequences, all conventional PCR (cPCR) assays were performed in duplicate in a Veriti 96 well Thermal Cycler (Life Technologies, Paisley, UK) using gDNA template when targeting intronless genes or cDNA template for spliced genes.

Each cPCR contained 10 μl of Fast Cycling PCR Master Mix (Qiagen), 2 pmol each of forward and reverse primer, 2 μl of nucleic acid template and nuclease-free water to a final reaction volume of 20 μl. Thermocycling was performed for five minutes at 95°C, followed by 40 cycles of 95°C for 15 seconds, 55°C for 30 seconds and 72°C for one minute.

Aliquots of each PCR product were mixed at a ratio of 5:1 with 6x Blue/Orange Loading Dye (Promega UK Ltd.) and then separated through electrophoresis in a 2% agarose gel containing SYBR^®^ Safe DNA Gel stain (Life Technologies) and visualized under ultraviolet light using a Universal Hood II transilluminator (Bio-Rad, Watford, UK). Predicted size DNA bands were cut from the gel and purified using the MinElute Gel Extraction Kit - Spin protocol (Qiagen) according to the manufacturer’s instructions. To elute DNA, 10 µL of buffer EB (10 mM Tris-HCl; pH 8.5) was added to the column membrane and after one minute, columns were centrifuged for one minute.

Purified DNA amplicons (in duplicate for each gene) were each subject to a single run of Sanger sequencing at the APHA Central Sequencing Unit. The resulting sequence data was assembled against predicted and published sequences using the Lasergene SeqMan Pro software (DNASTAR, Madison, WI, USA). The online NCBI BLAST tool was used to assess the percent identity of the assembled sequence of each gene to the corresponding genes of other species. Sequence alignments were performed using the Lasergene MegAlign software (DNASTAR).

#### 2.2.6 qPCR primer design

The mRNA sequence data for each Asian elephant gene described above, alongside sequence data generated for Asian elephant *IFNα* and *IFNβ* genes (39), were input into the online NCBI Primer-BLAST tool (42; https://www.ncbi.nlm.nih.gov/tools/primer-blast) with only three changes to the standard parameters: PCR product size was specified as 40 to 200 base pairs; Primer melting temperatures (T_m_) were specified as 58.0 to 62.0°C with an optimum value of 60.0°C; Primer size was specified as 15 to 30 bases with an optimum of 20 bases. Primers used for qPCR assay development are shown in **Table 2** and were synthesized by Eurofins Genomics. Published qPCR primers for the Asian elephant cytokines *IFNγ*, *IL10*, *IL12* and *TNFα* as described previously (43, 44) were also synthesized and included in this study.

**Table 2:** Asian elephant-specific qPCR primers.

Target Gene	F primer (5’-3’)	R primer (5’-3’)	Amplicon size (bp)
*CXCL10*	CTGCAAGTCTATCTTGCCCTCA	CATGCTTCTCTCTGCTTCCGA	160
*EF1α*	AGTCTGTTGAAATGCACCCC	GCTACATTGCCACGACGAAC	109
*IFNα*	GCTGAATGACCTGGAAGTCTGTTTG	CAGGCACAAGGGCTGTATTTCTTC	153
*IFNβ*	TGACAATCCTGGAGGAAAGTATGG	ATAGTCCTTCAGGTGGAGAATGG	80
*IFNγ*	GGAATATCTTAATGCAACTGATTCA	CCTGGTTGTCTTTCAAGTTGTCAA	157
*IFNω*	TTCGTGCAGGCAATGGAAGA	ACAACTTCCCAGGCACACTC	134
*IL1β*	GCTGGAATTTGAGTCAGCCG	TATTTCCCAGGAAGACGGGC	83
*IL6*	AAGGTTCATCCTCGCCGAAA	GCCTCCCTGCTGTTTTCACA	81
*IL8*	CTTGGCAGCTCTTGTGCTTT	GCATCGAAGTTCTGAAGCCAC	76
*IL10*	CCCTGGGGGAAAAGCTGA	CTCACGCATGGCTTTGTAGA	151
*IL12*	ATGCAAAGCTTTTGATGGACC	AAATTCAGGGCCTGCAT	91
*IRF3*	TGGGACCTTTTGTGGCAGAT	CGGAGGCATGTGGGAACTAC	154
*IRF7*	AGAGCCACCTTGGAGACGAT	CCACGGCAAGTATAGCTCCT	107
*IRF9*	CCTTCTTGCTTCAGGACCCC	TTCCACAGATGCCTGATCCC	200
*ISG15*	GGTCTCGAAGGAGATAGGCG	TCGCAGCTGTCCACTATCAG	141
*Mx1*	CAGTGGGGGAGAATCAGAGC	AATTCACAAAGCCTGGCAGC	168
*NFκB2*	CCGTGTTCCTCCAACTGAAA	CTCCTCCATAACCTCCAGCAGA	198
*OAS1*	GATCTGACGCTGACCTCGTG	GCGGGGGTTTTCCCATTTAC	170
*PKR*	AATTTGCTGCCAAACTCGCA	GTCACTGGGTGAAGTCACGA	101
*STAT1*	TCTACAGCAGGCTCGTCAGC	GGTGCGGTCCCATAACACTT	109
*STAT2*	TCAAACCAGAGCAACTGAGCA	ACAATGCACTCTCCGGGGTA	72
*TLR3*	ACTTGACCTTGGCCTGAACG	TTTTACAGGCCACCCTTCGG	176
*TLR9*	CTGCCCCCTACCCTAGACAA	GAAGGCCAAGTGATTGCCAC	72
*TNFα*	GAGATCCAAGTGACAAGCCTGTAG	TGAAGTTGCCCCTCGGTTT	66

#### 2.2.7 qPCR assay validation

All Asian elephant qPCR primers designed as part of this study were initially assessed for their ability to amplify the target gene from Asian elephant cDNA and to ascertain the analytical specificity of each assay. All qPCR assays were performed in a CFX96 Touch Real-Time PCR Detection System (Bio-Rad). Each reaction was performed in duplicate and contained 10 µL of 5x Brilliant III Ultra-Fast SYBR Green QPCR Master Mix (Agilent Technologies), 1 µL (2 pmol each) of forward and reverse primer, 2 µL of cDNA and nuclease-free water to a final volume of 20 µL. Reaction cycle conditions were ten minutes at 95°C followed by 40 cycles of 95°C for 30 seconds, 60°C for 30 seconds and 72°C for 30 seconds. A quantification plate read was taken after each cycle. A melting curve analysis was then completed from 60.0 to 95.0°C with a plate read after five seconds at each 0.5°C increment. Data from each qPCR assay run was analyzed using the CFX Manager 3.1 software (Bio-Rad) and samples were considered positive for the target gene if they produced a quantification cycle (Cq) value of less than 40 and produced a single melting curve peak at the expected temperature.

To confirm the efficacy of DNase treatment during RNA extraction and to confirm that assays did not produce false positive results, identical qPCR assays were carried out for eight of the qPCR primer pairs (*CXCL10*, *IRF3*, *IRF9*, *NFκB2*, *ISG15*, *TLR3*, *IFNα* and *IFNβ*) with extracted, non-reverse transcribed RNA as template.

Synthetic single-stranded DNA have been validated as alternative DNA standards for use in qPCR assays (45). Accordingly, synthetic DNA standards were synthesized by Eurofins Genomics for the newly designed Asian elephant qPCR assays, and for the qPCR primers of Landolfi et al. (43, 44) included in this study (shown in **Supplementary Table 1**). Each synthetic DNA standard contained the amplicon of a qPCR primer pair and an additional six bases of the relevant sequence at both the 5’ and 3’ ends, to improve primer binding. Sequences greater than 120 bases in length were appropriately truncated in the middle third, thereby preserving qPCR primer binding sites at each end. For each synthetic DNA standard, amplicon copy number per µL was calculated using the online EndMemo DNA/RNA copy number calculator tool (http://endmemo.com/bio/dnacopynum.php) and ten-fold serial dilutions in nuclease-free water were made to obtain a working range of 1.4 x 10^6^ to 1.4 x 10^0^ amplicon copies/µL. These dilution series were subsequently used as template in respective qPCR assay standard curves to calculate the efficiency and analytical sensitivity of each run of an individual assay.

### 2.3 Investigation of immunostimulants *in-vitro*

A subset of the validated qPCR assays was used to perform immune gene expression analyses investigating the *in-vitro* efficacies of the veterinary immunostimulants Zylexis, kindly provided by the veterinary department at Chester Zoo, and Zelnate, kindly provided by the manufacturer. In two independent experiments, aliquots of 150 µL of whole EDTA blood collected from five animals of varying ages from multiple institutions (shown in **Table 3**) were added to wells of three 96-well plates and mixed with either 50 µL of sterile phosphate buffered saline (PBS) or 50 µL of either Zylexis (Inactivated Parapoxvirus ovis, strain D1701, equivalent to a minimum of 23 IFN Units using the manufacturer’s standard preparation technique) or Zelnate. Both veterinary medicinal products were reconstituted according to the respective manufacturers’ instructions for veterinary *in vivo* administration. As commercial preparations protected by intellectual property patents, no further data is available regarding the precise concentrations of either iPPVO or bacterial plasmid DNA in either medicinal product, respectively. All blood samples were negative for EEHV1 by routine diagnostic qPCR (data not shown). Plates were covered and incubated for 2, 8 or 24 hours at 37°C, 5% carbon dioxide (CO_2_) on a microplate shaker set to 120 rpm.

**Table 3:** Details of Asian elephants included in the *in-vitro* immunostimulant studies.

Immunostimulant	Animal ID	Age (years)	Sex	Relatedness	Collection ID
Zylexis	1	33	Female		1
	2*	8	Female	Offspring of Animal 4	1
	3	19	Female		1
	4	35	Female		1
	5	35	Female		1
Zelnate	6	17	Male		2
	7	23	Female		3
	8	26	Female		4
	9	24	Female		4
	10*	3	Female	Offspring of Animal 9	4

*Juvenile animals.

#### 2.3.1 RNA extraction and cDNA synthesis

Samples (made to 250 µL with sterile PBS) were homogenized in 750 µL of Invitrogen™ TRIzol™ LS Reagent, as described above, and following lysis, 300 µL of the aqueous phase was transferred to a new tube.

Total RNA was extracted using the Applied Biosystems™ MagMAX™ *mir*Vana™ Total RNA Isolation Kit (Fisher Scientific) based on a modified version of the manufacturer’s protocol for urine samples. The volume of sample was 300 µL, the volume of isopropanol added was 300 µL and the volume of TURBO DNase™ Solution was 100 µL. The duration of the DNase incubation step was increased by 20 minutes and the volumes of the Rebinding Buffer and isopropanol added to each sample well during each run were increased to 100 µL and 200 µL, respectively. Following extraction, RNA samples were transferred to individual tubes and quantified using a Nanodrop 2000 spectrophotometer. RNA samples were stored at -80°C until further processing. First strand cDNA synthesis was performed in duplicate for each sample as described above using 150 ng of input RNA in each reaction.

#### 2.3.2 qPCR assays of *in-vitro* investigations

A subset of the qPCR assays was used to measure mRNA expression of *EF1α*, *CXCL10*, *IFNα*, *IFNβ*, *IL6*, *IL10*, *IRF7*, *ISG15*, *Mx1*, *NFκB2*, *OAS1* and *PKR*. Samples were tested in duplicate, and each run also included a standard curve for the target gene and a no-template control, also run in duplicate, to adjust for background fluorescence and to monitor for cross contamination. Standard curves and sample data, including melting curves, from each qPCR assay run were analyzed using the CFX Manager 3.1 software.

Quantification of fold-changes in gene of interest (GOI) expression between immunostimulated samples and PBS control samples was performed in Microsoft^®^ Excel^®^ for Office 365 (Microsoft) using the method described by Pfaffl (2001) (46) to account for discrepancies in primer efficiency. First, Cq values for technical replicates of each sample were averaged. In each experiment, the change in average Cq value (ΔCq) was calculated for each treated sample relative to the corresponding PBS control (ΔCq = Cq^PBS^ – Cq^immunostimulated^) for each GOI and the reference gene EF1α. The percent efficiency values (e) of each qPCR assay run calculated from the respective standard curves were converted (E) using the following formula: E = (e/100) + 1. Gene expression ratios (GER) of each GOI for each sample were calculated as GER = (E_GOI_)^ΔCqGOI^/(E_EF1α_)^ΔCqEF1α^. This represented the fold-change in GOI expression relative to PBS controls for each sample at each timepoint. To represent equivalent up- or down-regulation of gene expression, fold-changes were logarithmically transformed to base 2 (LOG2GER).

#### 2.3.3 Statistical analyses of *in-vitro* data

All statistical analyses were conducted according to guidance from Laerd Statistics (2015) (47) using IBM SPSS Statistics version 26 (IBM Corporation, Armonk, NY, USA). All GER datasets were tested for outliers and normality using box-and-whisker plots and Shapiro-Wilk tests respectively. To assess the suitability of EF1α as a reference gene within each experiment, paired-sample t-tests were performed to determine whether there was a statistically significant mean difference between *EF1α* Cq values following incubation in immunostimulant or PBS at each individual timepoint. For each experiment, paired-sample t-tests, Wilcoxon signed-rank tests or exact sign tests (depending on normality and symmetry of distributions) were performed to determine whether a statistically significant difference existed in mean (normal) or median (non-normal) LOG2GER between immunostimulated and control samples for each GOI at each timepoint. To statistically compare LOG2GER of a given GOI over the three timepoints, one-way repeated measures ANOVAs (including Mauchly’s test of sphericity) or Friedman tests (depending on normality and symmetry of distributions) were performed with Bonferroni-adjusted *post-hoc* pairwise comparisons. Where the assumption of sphericity was violated, a Greenhouse-Geisser correction was applied.

## 3 Results

### 3.1 Asian elephant mRNA sequences and qPCR assays

Sequences for 20 distinct Asian elephant genes were assembled using primers designed against the predicted sequence assemblies from Asian elephant genomes. Full mRNA coding sequences were determined for *CXCL10*, *GMCSF*, *IFNω*, *IL8*, *ISG15* and *TLR3* and partial coding sequences determined for all other genes (shown in **Table 4**).

**Table 4:** Asian elephant mRNA coding sequence data determined in this study.

Gene	Sequence length (bp)	GenBank accession number
CXCL10*	315	OR450021
*EF1α*	1377	OR450022
GMCSF*	405	OR450023
IFNω*	588	OR450030
*IL1β*	771	OR450031
*IL6*	677	OR450032
IL8*	318	OR450033
*IRF3*	772	OR450034
*IRF7*	709	OR450035
*IRF9*	814	OR450036
ISG15*	471	OR450037
*Mx1*	1340	OR450038
*NFκB2*	777	OR450039
*OAS1*	684	OR450040
*PKR*	1284	OR450041
*STAT1*	780	OR450042
*STAT2*	409	OR450043
TLR3*	2745	OR450044
*TLR9*	1770	OR450045

*Full coding regions.

bp, base pairs.

All Asian elephant gene sequences showed 98-100% nucleotide identity to the corresponding genes of the African elephant and 69-96% when compared to the respective human genes (shown in **Table 5**). Intriguingly, the full mRNA coding sequence for *IL8* generated in this study matched the predicted sequence of the African elephant (99.69%) more closely than the previously published data from Asian elephants (94.82%) (48). Accordingly, the corresponding Asian elephant IL8 protein had 99.05% amino acid identity to that of the African elephant protein but only 80.77% identity to that already published for the Asian elephant.

**Table 5:** Comparison of Asian elephant mRNA sequences obtained in this study.

Target Gene	Sequence length (bp)	*E.maximus*	Predicted *L.africana*	*H.sapiens*
*CXCL10*	315		100.00	83
*EF1α*	1377		98.18	94
*GMCSF*	405		99.39	75
*IFNω*	588		98.64	77
*IL1β*	771	99.74*	99.61	79
*IL6*	677		99.70	80
*IL8*	318	94.82*	99.69	77
*IRF3*	772		99.87	81
*IRF7*	709		98.30	69
*IRF9*	814		98.77	80
*ISG15*	471		99.79	74
*Mx1*	1340		99.40	81
*NFκB2*	777		99.74	90
*OAS1*	684		99.42	78
*PKR*	1284		99.69	75
*STAT1*	780		99.74	96
*STAT2*	409		100.00	89
*TLR3*	2745		99.49	85
*TLR9*	1770		99.55	84

bp, base pairs.

*Comparison to previously published Asian elephant sequences.

Percentage (%) identity to corresponding mRNA sequences of other species is shown for comparison.

### 3.2 qPCR assay validation

Initially, primer pairs were assessed for their ability to amplify Asian elephant DNA targets, producing a single melting curve peak at the expected temperatures. Synthetic single-stranded DNA standards were used successfully to produce calibration curves and therefore calculate the qPCR efficiency and analytical sensitivity for each assay (shown in **Table 6**). Assay efficiencies varied between 89 and 107% and the limit of detection (LoD), defined as the lowest concentration at which at least 95% of replicates produced the expected single qPCR melting curve peak, varied between 28 and 280 DNA copies per reaction.

**Table 6:** Asian elephant qPCR assay efficiencies and limits of detection (LoD) as used in this study.

Target Gene	Standard curve slope	Efficiency (%)	y int.	R^2^	LoD (copies per reaction)
*CXCL10*	-3.604	89.4	42.679	0.995	28
*EF1α*	-3.548	91.4	43.119	0.997	280
*IFNα*	-3.375	97.8	41.633	0.979	28
*IFNβ*	-3.404	96.7	41.209	0.989	280
*IFNγ*	-3.616	89.0	38.972	0.998	280
*IFNω*	-3.295	101.1	41.616	0.979	280
*IL1β*	-3.308	100.6	39.896	0.990	28
*IL6*	-3.177	106.4	39.634	0.990	280
*IL8*	-3.482	93.7	40.864	0.996	280
*IL10*	-3.334	99.5	36.140	0.980	28
*IL12*	-3.375	97.8	34.440	0.979	28
*IRF3*	-3.289	101.4	41.281	0.988	280
*IRF7*	-3.389	97.3	41.404	0.998	280
*IRF9*	-3.485	93.6	42.352	0.991	280
*ISG15*	-3.349	98.9	42.102	0.983	280
*Mx1*	-3.318	100.1	41.720	0.989	280
*NFκB2*	-3.319	100.1	41.630	0.979	280
*OAS1*	-3.494	93.3	40.851	0.998	280
*PKR*	-3.390	97.2	40.795	0.994	28
*STAT1*	-3.344	99.1	41.030	0.983	280
*STAT2*	-3.395	97.0	40.256	0.995	280
*TLR3*	-3.495	93.2	41.461	0.997	280
*TLR9*	-3.398	96.9	38.935	0.999	28
*TNFα*	-3.397	97.0	36.124	0.995	28

All standard curve data was calculated using CFX Manager 3.1 software. The limit of detection (LoD) represents the lowest detectable copy number of target amplicon in a 20 µL reaction.

### 3.3 Gene expression analysis of immunostimulation

To assess the level of immunostimulation induced in Asian elephant whole blood, qPCR assays described above were used to measure mRNA expression of *EF1α*, *CXCL10*, *IFNα*, *IFNβ*, *IL6*, *IL10*, *IRF7*, *ISG15*, *Mx1*, *NFκB2*, *OAS1* and *PKR*, following *in-vitro* addition of Zylexis or Zelnate. Asian elephant mRNA of all targeted genes was successfully detected in all qPCR assays and in almost all samples. Non-specific amplification was not recorded in any no-template controls and synthetic DNA standards successfully generated calibration curves in all assays. All GER datasets followed normal distributions as assessed by Shapiro-Wilk tests (p>0.05). Mean differences in *EF1α* Cq values did not differ between stimulated and control samples at any timepoint in either experiment (p≥0.05), confirming *EF1α* as a suitable reference gene.

**Figure 1:**
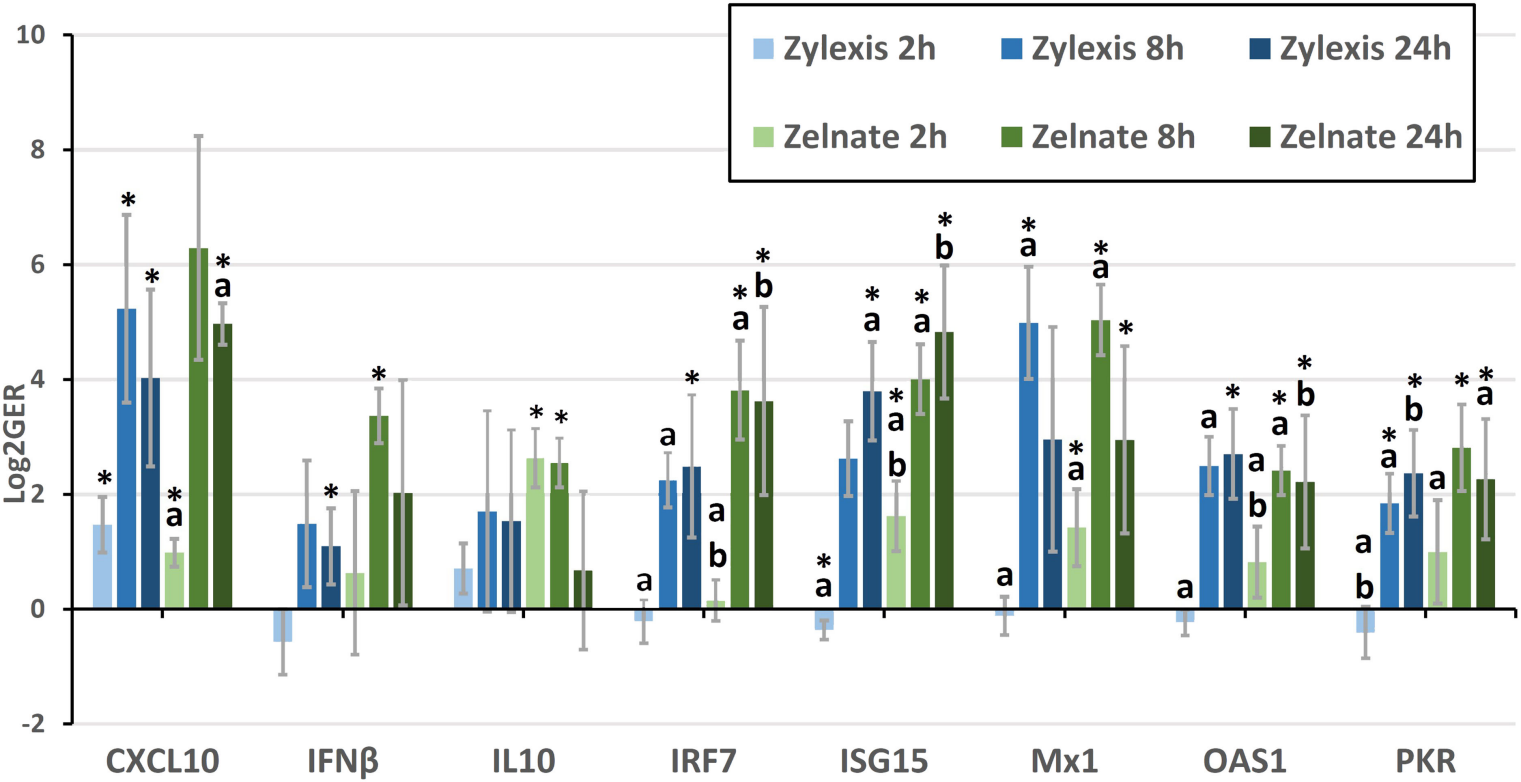
Mean logarithmically transformed fold-changes (LOG2GER) of *CXCL10*, *IFNβ*, *IL10*, *IRF7*, *ISG15*, *Mx1*, *OAS1*, and *PKR* following immunostimulation with either Zylexis or Zelnate. Error bars represent the 95% confidence interval of the mean. Statistically significant differences (p<0.05) compared to PBS controls are indicated by asterisks. Statistically significant differences (p<0.05) between timepoints for the same gene, for the same medication are indicated by letters. Data shown is related to that represented in **Table 7**.

Geometric means of fold-changes in gene expression are shown in **Table 7** for all animals and for all target genes within each experiment. Both immunostimulant medications produced greater than four-fold increases in relative expression of *CXCL10*, *IL6*, *IRF7*, *ISG15*, *Mx1*, *OAS1* and *PKR* during either experiment. Only Zelnate produced a greater than four-fold increase in relative expression of *IFNβ* and *IL10* and greater than two-fold increases in relative expression of *IFNα*. Neither immunostimulant medication produced greater than three-fold increases in relative expression of *NFκB2*. Where samples failed to demonstrate specific amplification of a given target gene, missing data points were excluded from subsequent statistical analyses. Mean LOG2GER for all animals, and specifically for target genes that demonstrated greater than four-fold increases in relative expression, are displayed graphically in **Figure 1**.

**Table 7:** Geometric means of normalised fold-changes in gene expression following immunostimulation with either Zylexis or Zelnate.

Medication	Timepoint	*CXCL10*	*IFNα*	*IFNβ*	*IL6*	*IL10*	*IRF7*	*ISG15*	*Mx1*	*NFκB2*	*OAS1*	*PKR*
**Zylexis**	**2h**	2.77	0.18 ^a^	0.67	386.44*	1.63	0.86 ^a^	0.78 ^a^	0.92 ^a^	1.05 ^a^	0.85 ^a^	0.75 ^a b^
	**8h**	37.56	0.26	2.79	3.62^#^	3.25	4.74 ^a^	6.14	31.66^+ a^	2.71 ^a^	5.63 ^a^	3.59 ^a^
	**24h**	16.28	1.13 ^a^	2.13	16.38*	2.90	5.61	13.88 ^a^	7.76^+^	2.37	6.50	5.17 ^b^
**Zelnate**	**2h**	1.97 ^a^	0.71	1.55	5.32	6.21	1.11 ^a b^	3.08 ^a b^	2.67 ^a^	1.04	1.76 ^a b^	1.99 ^a^
	**8h**	78.17*	2.82	10.33	11.70^#^	5.84	14.05 ^a^	16.05 ^a^	32.81 ^a^	1.86	5.33 ^a^	7.02
	**24h**	31.31* ^a^	1.13	4.06	3.94^#^	1.60^+^	12.32 ^b^	28.33 ^b^	7.72	2.01	4.63 ^b^	4.80 ^a^

^+^n=4; *n=3; ^#^n=2.

^a,b^statistically significant (p<0.05) differences between timepoints for the same gene, for the same medication.

Other than where indicated, n=5. Statistically significant differences (p<0.05) compared to PBS controls were determined using the logarithmically transformed fold-change (LOG2GER) datasets and are indicated by shaded cells.

LOG2GERs following incubation with Zylexis were statistically significant (p<0.05), compared to PBS controls, for *CXCL10* at all three timepoints; for *IFNα* at both 2h and 8h; *IFNβ*, *IRF7* and *OAS1* at 24h; *IL6* at 2h; *ISG15* at both 2h and 24h; *Mx1* and *NFκB2* at 8h; and *PKR* at both 8h and 24h. Following incubation with Zelnate, LOG2GERs were statistically significant (p<0.05) compared to controls for *ISG15* and *Mx1* at all three timepoints; *CXCL10* at both 2h and 24h, *IFNβ* and *NFκB2* at 8h; *IL6* at 2h; *IL10* at both 2h and 8h; and *IRF7*, *OAS1* and *PKR* at both 8h and 24h.

Statistically significant (p<0.05) differences in LOG2GERs following incubation with Zylexis were found between 2h and 8h for *IRF7*, *Mx1*, *NFκB2*, *OAS1* and *PKR* and between 2h and 24h for *IFNα*, *ISG15* and *PKR*. Following incubation with Zelnate, statistically significant (p<0.05) differences in LOG2GERs were found between 2h and 8h for *IRF7*, *ISG15*, *Mx1* and *OAS1* and between 2h and 24h for *CXCL10*, *IRF7*, *ISG15*, *OAS1* and *PKR*. No statistically significant differences in LOG2GERs were seen for any GOI following incubation with either immunostimulant medication between 8h and 24h.

Statistical comparison was not possible for LOG2GERs of the juveniles, of which there was only one included in each experiment (**Table 8**). Either immunostimulant medication resulted in greater than four-fold increases in expression of *CXCL10*, *IFNβ*, *IRF7*, *ISG15* and *Mx1*. Only Zylexis produced greater than four-fold increases in relative expression of *IL6*, *OAS1* and *PKR*. Neither immunostimulant medication produced greater than a two-fold increase in relative expression of *IFNα*.

**Table 8:** Fold-changes in normalised gene expression ratios in blood from two individual juvenile Asian elephants following immunostimulation with either Zylexis (n=1) or Zelnate (n=1).

Medication	Timepoint	*CXCL10*	*IFNα*	*IFNβ*	*IL6*	*IL10*	*IRF7*	*ISG15*	*Mx1*	*NFκB2*	*OAS1*	*PKR*
**Zylexis**	**2h**	4.06	0.24	1.55	883.15	1.66	1.08	0.88	1.20	1.47	1.04	0.97
	**8h**	10.84	0.22	4.16	2.42	3.31	4.49	7.94	23.30	2.50	6.87	2.51
	**24h**	8.18	1.23	1.48	2.16	1.06	3.70	15.10	1.28	1.52	6.70	7.24
**Zelnate**	**2h**	2.73	1.16	1.97	3.74	3.44	0.71	1.54	1.13	0.66	0.91	1.34
	**8h**	78.01	1.52	13.10		3.24	7.06	8.32	16.61	1.63	3.44	3.26
	**24h**	36.17	1.16	1.42		0.51	3.15	9.90	1.43	0.98	1.53	2.09

IL6-specific cDNA failed to amplify in the 8h and 24h samples from the juvenile’s blood incubated with Zelnate, precluding fold-change calculations. Data shown is also included in **Table 7**.

## 4 Discussion

This study was performed to evaluate the immune response of Asian elephant blood cells following incubation with two authorized veterinary medicinal products, both indicated for use when an innate immune response is needed.

Genetic data was limited at the start of the study as the draft genome for Asian elephants had not yet been assembled, and only a limited number of Asian elephant mRNA sequences were deposited in GenBank. Of these, just 11 mRNA sequences were directly related to the immune system (43, 44, 48–50), and the majority were relevant in adaptive immunity.

The sequence data generated was derived from a single animal and the detection of polymorphisms across the available Asian elephant gene sequences is interesting, but not unexpected. For example, a disparity was observed between the *IL8* sequence determined here and the one previously described (48). This can be explained by a deletion and insertion, both identified in the published mRNA sequence at positions 65 and 92 that results in a mismatch between amino acids 22 and 30 (data not shown). Given the near complete match with the predicted *IL8* sequence of African elephants (99.1% at the aa level), and the similarity observed for other species, it is reasonable to conclude that the sequence reported here is correct.

Zylexis and Zelnate are already authorized as veterinary immune modulators. Given the reported benefits in other species (14, 21, 23, 25, 51–53) it was warranted to analyze and compare the effects of both on Asian elephant blood cells *in-vitro*, by measuring gene expression in response to stimulation using a relevant subset of the qPCR assays validated in the current study, with emphasis on the innate immune system.

It is not clear why mRNA for *CXCL10*, *IL6*, *IL10* and *Mx1* could not be detected in a limited number of samples from different animals, at different timepoints, for either immunostimulant medication, leading to their exclusion from gene expression analysis, as indicated in **Table 7**. However, low target gene expression is the most likely explanation. Given the degree of analytical specificity demonstrated by each qPCR primer pair during assay validation, it is reasonable to exclude suboptimal primer specificity as an underlying cause for these results. Furthermore, as the same cDNA template material was used across multiple qPCR runs, targeting a number of genes, the presence of PCR inhibitors is also highly unlikely.

An expression of type I IFNs within 24 hours of the administration of, or incubation with, such immunostimulants had been demonstrated in previous studies (51, 54–56), but data from the current study demonstrated a more complex picture. Firstly, the overall induction of *IFNβ* expression was significantly stronger than for *IFNα*. Since the latter is strongly associated with the biology of plasmacytoid dendritic cells (pDC) (57–60) it may well be that pDC viability was reduced in the blood samples, possibly due to transport at ambient temperature. Furthermore, it is notable that the induction of *IFNβ* was significantly stronger following *in-vitro* incubation with Zelnate than with Zylexis. The level of *IFNβ* gene expression and the differences between Zelnate and Zylexis correlate well with the upregulation of IRF7, a central transcription factor for type I IFNs (61–63).

Furthermore, it is notable that *ISG15* and the effector protein *Mx1* were upregulated to a greater degree than the IFN-related effector genes *OAS1* and *PKR*. Additionally, the upregulation of *ISG15* was more marked following incubation with Zelnate, and the level of expression of this gene has previously correlated with innate anti-viral protection (64, 65).

The upregulation of *IL6* mRNA as a pro-inflammatory marker, alongside *CXCL10*, was expected, in line with results reported for bovine (66), porcine (67) and equine PBMCs (56). However, it must be noted that the *IL6* mRNA expression at 2h from all samples treated with Zylexis were skewed by an outlier, corresponding to a juvenile animal. Therefore, the *IL6* gene expression data presented here should be interpreted with caution. Additionally, neither medication induced a high induction of *NFkB2*.

*IL10*, which can act as a delayed immune-regulatory protein (16, 17, 19, 52) was not significantly induced by either medicinal product and more importantly, *IL10* expression did not appear to be sustained over the 24 hours investigated, contrary to published results from other species (14).

For an accurate interpretation, it must be acknowledged that mRNA expression is not synonymous with protein production (68–70). Accordingly, minor changes in the expression of potent mediators, such as cytokines, can potentially have far-reaching biological effects (71–74). Conversely, what may be calculated as statistically significant changes in gene expression may produce relatively negligible downstream consequences due to post-transcriptional and post-translational regulatory mechanisms (75, 76). However, previous studies have demonstrated a generally close relationship between innate immune gene expression and corresponding protein production (16, 17, 19, 56, 66). Therefore, the *in-vitro* results observed in the current study demonstrate the potential benefits of these medications in the stimulation of the innate immune system in Asian elephants.

Additionally, the ambient temperatures at which blood samples were stored from the point of collection and importantly, during overnight transport to the laboratory, must be taken into account. This will undoubtably have had an impact on immune cell viability. The results obtained here, however, still reflect the immunostimulatory potential of both Zylexis and Zelnate in Asian elephants, which is therefore likely to have been underestimated in this study.

While the inclusion of only a single juvenile elephant in each experiment precludes more meaningful comparison to the adults investigated, a significant majority of datapoints obtained from these samples sit close to the geometric means of all animals. Accordingly, it seems reasonable to assume that the innate immune system of juvenile Asian elephants is not substantially different from that of adults.

The significant upregulation of *CXCL10*, *ISG15*, *Mx1*, *OAS1* and *PKR* within 24 hours of incubation with either of the immunostimulant products demonstrated the stimulation of key antiviral effector pathways. Alongside the more immediate anti-viral effects, stimulating the innate immune system to control a primary infection could also trigger the development of longer-lasting protection by enhancing the concurrent adaptive immune response. This suggests a beneficial effect could potentially be achieved following clinical administration in cases of early EEHV infection, or even during escalating viraemia. An affirming outcome of the work described here is presented in a previous case report (38), where Zelnate, in conjunction with the administration of human interferon products and more general supportive measures, appears to have contributed to an elephant calf’s full clinical recovery within two weeks. With the eventual aim of applying immunostimulant medications in such a clinical setting, we focused on the two iPPVO and CpG DNA motif containing immunostimulants, respectively, in this study as they are already authorised for veterinary species in Europe. However, further studies will be required to determine the more precise effects that these medications, and other immunostimulants with the potential for use in Asian elephants, may have in the protection of juvenile animals against EEHVs, including their influence on the generation of a longer-lasting adaptive immune response against these viruses.

## Data availability statement

The datasets presented in this study can be found in online repositories. The names of the repository/repositories and accession number(s) can be found in the article/**Supplementary Material**.

## Ethics statement

Ethical approval was not required for the studies involving animals in accordance with the local legislation and institutional requirements because all samples used in this study were collected in accordance with the Veterinary Surgeons Act 1966, in that they were collected for the sole purpose of ensuring the health and welfare of the animals involved. This was in full accordance with routine veterinary practice, defined by section 25.7 of the Supporting Guidance of the Royal College of Veterinary Surgeons (RCVS) Code of Professional Conduct. The experimental procedures described in this manuscript subsequently used only the sample material remaining after all clinical purposes had been fulfilled, and with the informed consent of both the responsible veterinarian and the relevant institution involved. Written informed consent was obtained from the owners for the participation of their animals in this study

## Author contributions

JH: Conceptualization, Data curation, Formal analysis, Investigation, Methodology, Project administration, Writing – original draft, Writing – review & editing. TM: Investigation, Methodology, Project administration, Supervision, Writing – review & editing. AD: Conceptualization, Funding acquisition, Methodology, Project administration, Resources, Supervision, Visualization, Writing – review & editing. FS: Conceptualization, Funding acquisition, Methodology, Project administration, Resources, Supervision, Visualization, Writing – review & editing.
