# Supplementary material for: Immunostimulation of Asian elephant (Elephas maximus) blood cells by parapoxvirus ovis and CpG motif-containing bacterial plasmid DNA upregulates innate immune gene expression
Source: Front Immunol. 2024 Mar 25;15:1329820. doi: 10.3389/fimmu.2024.1329820 (PMC10999609; doi:10.3389/fimmu.2024.1329820)
Supplement: Supplementary file 2 [file Table_1.docx]

Supplementary Material

Supplementary table 1. Asian elephant specific qPCR synthetic single-stranded DNA standards.

| **Gene** | **Synthetic oligonucleotide standard** | **Length (bases)** |
| --- | --- | --- |
| *CXCL10* | TGA TTC CTG CAA GTC TAT CTT GCC CTC ATG TTG AGA TCA TTG TCA CAA TGA AAA AAA GTG GCC ATC AGA AAG GAA AGG TCT ACA ATG TCT CCT CGG AAG CAG AGA GAA GCA TGA TAA GCA | 120 |
| *EF1α* | AAG TAA AGT CTG TTG AAA TGC ACC CCG AAG CTT TGA GCG AAG CTC TCC CTG GGG ACA ATG AGG CTT CAA TGT CAA GAA TGT CTC TGT CAA AGA TGT TCG TCG TGG CAA TGT AGC TGG TGA | 120 |
| *IFNα* | TCA GCA GCT GAA TGA CCT GGA AGT CTG TTT GAT GCA GGA GAT GGG GGT AGA AGA AAC TCC CTT CCA AAG AAT CAC TGT CTA TCT GAC AGA GAA GAA ATA CAG CCC TTG TGC CTG GGA GAT | 120 |
| *IFNβ* | GTC TGG TGA CAA TCC TGG AGG AAA GTA TGG AGG AGG AGA ACC CCA CCT CGA GAA ACA TCA TGA CCA TTC TCC ACC TGA AGG ACT ATT ACT TA | 92 |
| *IFNγ* | GGAATATCTTAATGCAACTGATTCAGATATAGATATTTTGAAGAACTGGAAAGAGGAGAGTGACATAGTTCATAGTTTCCTTTTACCTCAAAATCTTTGACAACTTGAAAGACAACCAGG | 120 |
| *IFNω* | ACC TGC TTC GTG CAG GCA ATG GAA GAG GAA AAA TCT GTC CTG CCC ATT GAG GCC CCT GAA AAT CTG TTC CTA TCT GAA AGA GAA GGA ATA CAG TGA GTG TGC CTG GGA AGT TGT CAG AGT | 120 |
| *IL1β* | AGA CAA GCT GGA ATT TGA GTC AGC CGA ATA CCC CAA CTG GTA TAT CAG CAC TTC TCA AGT GGA AGA AAT GCC CGT CTT CCT GGG AAA TAC CAG AG | 95 |
| *IL6* | ACT CAT AAG GTT CAT CCT CGC CGA AAT CTC TGT TCT GAG AAA GAA GAT GTG TGA CAA ATA TGA CAA GTG TGA AAA CAG CAG GGA GGC ACT GGC | 93 |
| *IL8* | TGC TCT CTT GGC AGC TCT TGT GCT TTC TGC AGC TCT CTG CAA AGC TGC AGT TTT GCC AAT GGT GGC TTC AGA ACT TCG ATG CCA GTG T | 88 |
| *IL10* | CCCTGGGGGAAAAGCTGACGACCCTCAGGACCGCTGTCACCGATTTCTTCCCTGTGAAAATAAGAGCAAAGGTAAGCTCCAAGAGAAAGGTGTCTACAAAGCCATGCGTGAG | 112 |
| *IL12* | ATGCAAAGCTTTTGATGGACCCCAAGAGGCAGATCTTTCTGGATCAAGACATGTTGGCAGCTATTGACAAGCTGATGCAGGCCCTGAATTT | 91 |
| *IRF3* | TCA ACC TGG GAC CTT TTG TGG CAG ATC TGA TTG CCT TCA TCG AAG GAA GCA AAC GCT CAC CCA GCC GTG GAC CAA GAA GCT GGT GAT GGT CAA GGT AGT TCC CAC ATG CCT CCG GGT CCT | 120 |
| *IRF7* | TGC AGC AGA GCC ACC TTG GAG ACG ATT TGC TGG AAG CAG CAT GGA GGA TGG ATC CGG CCC CCC AGC AGC CTT CTG GCC CAG TGC TGG CTT GTG AGG AGC TAT ACT TGC CGT GGG CAG TG | 119 |
| *IRF9* | TCA GCC CCT TCT TGC TTC AGG ACC CCT TCA ATA ATG AGA GGG GAA CCA GTG GGG GAG CAG GAA GGC ACA GAC ACA ACT GAG GCC CCC TTT GAA GGG GAT CAG GCA TCT GTG GAA TTC GTG | 120 |
| *ISG15* | GCA GCG GGT CTC GAA GGA GAT AGG CGT GCC TAC CTT CCA GCT GCG GCT GGC CCA TTC AAA GGA CCA GAG CCT GGG CCC TGG CAG CAT GGT CCT GCT GAT AGT GGA CAG CTG CGA GCC CCT | 120 |
| *Mx1* | AAG AGT CAG TGG GGG AGA ATC AGA GCC GGC TAT TTA CAA AAA TCC GAA GTG AGT TCC TCA TTT CGG AAT TTG AAA ATC AGT ATC GTG GCA GAG AGC TGC CAG GCT TTG TGA ATT TTC GGA | 120 |
| *NFκB2* | CTG TCA CCG TGT TCC TCC AAC TGA AAC GCA AGC GTG GGG GGG ATG TCT CTG ACT CCA AAC GCT CCC ACA TGG GTG GAG GCT CTG GAG GCT CTG CTG GAG GTT ATG GAG GAG CTG TAA GCA | 120 |
| *OAS1* | AAG GCC GAT CTG ACG CTG ACC TCG TGG TCT TCC TCA GCA ATC TCA CAA GTT TTC AGG AGC GAA GAA GAG TTT GAC GTG GAA TTT GAG GTC CAG AGT AAA TGG GAA AAC CCC CGC GTG CTT | 120 |
| *PKR* | CAA AAC AAT TTG CTG CCA AAC TCG CAT ATG ATG AGA TAA CAG GAG AAA AAA CAT CAG AGA AAG CTG ACT CAG CAT CGT TTG GTT CTT TCG TGA CTT CAC CCA GTG ACT CCA GT | 113 |
| *STAT1* | GGA GAG TCT ACA GCA GGC TCG TCA GCA GCT CAA AAA GCT TGA GGA GCT GGA ACA GAA GTA ACC TAC GAG CAT GAC CCC ATC ACA AAA AAC AAA CAA GTG TTA TGG GAC CGC ACC TTC ACT | 120 |
| *STAT2* | GAG GCC TCA AAC CAG AGC AAC TGA GCA TGC TGA GGG ACA AAT TAT TTG GGC AGA ACT CTA CCC CGG AGA GTG CAT TGT CCT GGG | 84 |
| *TLR3* | AGA GGT ACT TGA CCT TGG CCT GAA CGA AAT TGG GCA AGA ACT CAC AGG CCA GGA ATG GAG TTG CCT TGG TTC CAA GCC TTC AAC AAC TGA TGC TCC GAA GGG TGG CCT GTA AAA ATG TGG | 120 |
| *TLR9* | ACG CTC CTG CCC CCT ACC CTA GAC AAC CTC CCC AAG AGC TTG CAG CTG CTG CGA CTC CGT GGC AAT CAC TTG GCC TTC TTC AAC | 84 |
| *TNFα* | GAGATCCAAGTGACAAGCCTGTAGCACACGTTGTAGCAAACCCCCAAACCGAGGGGCAACTTCA | 64 |
